# Questioning risk compensation: pre-exposure prophylaxis (PrEP) and sexually transmitted infections among men who have sex with men, capital region of Denmark, 2019 to 2022

**DOI:** 10.2807/1560-7917.ES.2024.29.13.2300451

**Published:** 2024-03-28

**Authors:** Sebastian von Schreeb, Susanne Kriegel Pedersen, Hanne Christensen, Kristina Melbardis Jørgsensen, Lene Holm Harritshøj, Frederik Boetius Hertz, Magnus Glindvad Ahlström, Anne-Mette Lebech, Suzanne Lunding, Lars Nørregaard Nielsen, Jan Gerstoft, Gitte Kronborg, Frederik N Engsig

**Affiliations:** 1 Department of Infectious Diseases, Copenhagen University Hospital – Amager and Hvidovre, Copenhagen, Denmark; 2 Department of Infectious Disease Epidemiology and Prevention, Statens Serum Institut, Copenhagen, Denmark; 3 Department of Infectious Diseases, Copenhagen University Hospital – Rigshospitalet, Copenhagen, Denmark; 4 Department of Clinical Microbiology, Copenhagen University Hospital - Amager and Hvidovre, Copenhagen, Denmark; 5 Department of Clinical Immunology, Copenhagen University Hospital – Rigshospitalet, Copenhagen, Denmark; 6 Department of Clinical Medicine, University of Copenhagen, Copenhagen, Denmark; 7 Department of Clinical Microbiology, Copenhagen University Hospital – Rigshospitalet, Copenhagen, Denmark; 8 Department of Clinical Microbiology, Copenhagen University Hospital - Herlev and Gentofte, Copenhagen, Denmark; 9 Department of Internal Medicine, Copenhagen University Hospital – Herlev and Gentofte, Copenhagen, Denmark; 10 Department of Pulmonary and Infectious Diseases, Copenhagen University Hospital – North Zealand Hospital, Copenhagen, Denmark

**Keywords:** Pre-exposure Prophylaxis, PrEP, Gonorrhoea, Gonorrhea, Chlamydia, Syphilis, HIV, risk compensation

## Abstract

**Background:**

Pre-exposure prophylaxis (PrEP) effectively prevents HIV, but its association with sexually transmitted infections (STIs) has raised concerns about risk compensation, potentially impacting the expansion of PrEP programmes.

**Aim:**

We examined the relationship between PrEP and the incidence of chlamydia, gonorrhoea and syphilis.

**Methods:**

In this prospective cohort study, we compared STI rates before and after PrEP initiation among users in the capital region of Denmark (2019–2022), calculating incidence rate ratios adjusted for age and testing frequency (aIRR). To pinpoint when increases began, we plotted weekly STI rates, adjusting the timeline to correspond with PrEP initiation.

**Results:**

The study included 1,326 PrEP users with a median age of 35 years. The STI incidence rate per 100,000 person-years rose from 35.3 before to 81.2 after PrEP start, with an aIRR of 1.35 (95% CI: 1.18–1.56). Notably, this increase preceded PrEP initiation by 10–20 weeks. Specific aIRR for chlamydia, gonorrhoea and syphilis were 1.23 (95% CI: 1.03–1.48), 1.24 (95% CI: 1.04–1.47) and 1.15 (95% CI: 0.76–1.72), respectively. In subanalyses for anatomical sites aIRR was 1.26 (95% CI: 1.01–1.56) for rectal chlamydia and 0.66 (95% CI: 0.45–0.96) for genital gonorrhoea.

**Conclusion:**

We found a 35% increase in STI incidence associated with PrEP use. It started before PrEP initiation, challenging the assumption that PrEP leads to risk compensation. Instead, the data suggest that individuals seek PrEP during periods of heightened sexual risk-taking. Consequently, PrEP programmes should include sexual health consultations, STI testing, treatment and prevention strategies to prevent HIV and improve sexual health.

**Key public health message**

**What did you want to address in this study and why?**

Pre-exposure prophylaxis (PrEP) is a pill taken daily which effectively prevents HIV infection among high-risk populations such as men who have sex with men. Previous work has found an association between PrEP use and sexually transmitted infections (STI), which has been interpreted as sexual risk compensation. However, it has not been readily investigated if PrEP use leads to increased sexual risk-taking or if sexual risk-taking leads people to PrEP?

**What have we learnt from this study?**

Comparing rates for chlamydia, gonorrhoea and syphilis before and after PrEP initiation, the current study found a significant association between incidence of these important STI and PrEP use. However, the increase in STI preceded PrEP use, which questions the previous assumption of sexual risk compensation.

**What are the implications of your findings for public health?**

These findings have implications for clinicians and policymakers who take decisions on the extension of PrEP programmes. The prevailing idea of sexual risk compensation may have hindered a wider implementation of PrEP initiatives. The findings in the current study question that idea and instead indicate that people seek PrEP at times of sexual risk-taking, which is when it is most needed.

## Introduction

Pre-exposure prophylaxis (PrEP) is the use of antiviral drugs to protect high-risk populations from acquiring human immunodeficiency virus (HIV) [1] and has shown effectiveness above 99% [2]. Since their recommendation by the World Health Organization in 2015 [3], PrEP programmes have been adapted by more than 144 countries, providing protection to millions of people worldwide [4].

Despite the success in minimising the risk of HIV infection, there is a concern that PrEP use could be related to increases in bacterial sexually transmitted infections (STI) such as chlamydia, gonorrhoea and syphilis. During the years of PrEP roll-out, condom use has declined among high-risk groups, and STI incidence has increased [5]. It remains unclear whether these changes are directly linked to PrEP use or are caused by other factors [6].

Studies exploring the association between PrEP use and STIs often refer to the concept sexual risk compensation [7,8]. Superficially, risk compensation could be understood as the notion that people are more cautious when facing high risks, and less cautious when facing low risks. If PrEP lowers the fear of HIV, people may relax their safety practices. However, the definition of risk compensation, rooted in the psychological theory of risk homoeostasis, extends beyond this straightforward notion. Risk compensation means that any reduction in risk will be completely counterbalanced by riskier behaviour, maintaining an individual's risk set point [9]. As a consequence, preventive interventions will be inherently ineffective [10]. There is a scientific debate about sexual risk compensation, i.e. that PrEP will cause sexual risk-taking, counteracting its preventive effect. The concept of sexual risk compensation has been criticised for lacking evidence, relying on oversimplified models of human behaviour and inducing moral hysteria [9,11,12], but the term is still commonly used as an argument against PrEP [7,13].

Studies exploring the empirical foundation for sexual risk compensation related to PrEP use have reported mixed results. A systematic review on STI incidence, incorporating findings from open-label PrEP studies, suggested that early studies found no signs of risk compensation, while later studies found partial support for an increase in STIs associated with PrEP use [14]. In parallel, two other reviews exploring changes in condom use related to PrEP initiation showed inconsistent results [15,16], with condoms expected to provide substantial, but not absolute, protection against STIs. It is important to consider the biases present in the different types of studies. The early randomised trials may not have captured behavioural changes related to PrEP if participants were uncertain or, in some trials, not aware of whether they were protected against HIV or not. Observational studies, however, often rely on aggregate and/or self-reported retrospective data and are not randomised. As a result, these studies struggle to separate cause and effect, leaving it unclear whether PrEP use increased unprotected sex or if changes in sexual risk-taking lead people to PrEP [15].

In Denmark, the combination of a unique civil registration (CPR) number and comprehensive databases of microbiological test results allows for high-quality data on STI incidence both before and after PrEP initiation. This data infrastructure makes it possible to investigate the timing of changes in STI incidence in relation to PrEP initiation, which is useful when investigating possible causality.

Furthermore, to better understand the association between STI incidence and PrEP use, it is important to identify individual factors predicting changes in STI rates for people starting PrEP, so that individualised preventive efforts can be made [14]. It is well known that STIs are more common among younger individuals [16], but it is unclear if changes in STI incidence occur more among younger or older PrEP users. Some evidence from Amsterdam suggests a larger increase in condomless sex among older PrEP users [17], although a later follow-up study saw no effect on STI incidence [18]. A within-participant design comparing STI incidence before and after PrEP start is required to analyse a connection between risk behaviour and age [14].

Changes in STI incidence may also depend on the anatomical site of the infection. Oral sex is the most common sex act among men who have sex with men, and condom use for oral sex is rare [19,20]. Consequently, if PrEP users were less likely to use condoms, any change in STI incidence would be more pronounced for anal and genital STIs than oropharyngeal STIs. Along these lines, a smaller study from Montreal found a significant increase in anal and genital chlamydia after PrEP start, while no change was seen for oropharyngeal chlamydia [21].

The scientific debate about sexual risk compensation is a central challenge to the extension of PrEP programmes globally [7]. If PrEP use causes higher rates of chlamydia, gonorrhoea and syphilis, efforts to combat such a side effect must be made. If, on the other hand, the claims of risk compensation are unfounded, the concept may hamper the development of PrEP programmes, thereby failing to protect people at risk from HIV. To scrutinise the sexual risk compensation concept in a Danish context, the current study used a within-participant method to examine changes in STI incidence before and after PrEP start in the capital region of Denmark. Our study set out to answer the following questions: Is PrEP use associated with an increased incidence of chlamydia, gonorrhoea and syphilis? How does the timing of any changes in STI incidence rates correspond to the initiation of PrEP? Is there a larger change in incidence for anal STIs, as compared with oral or genital infections? Are there age differences in PrEP-related changes in STI incidence?

## Methods

In a cohort of PrEP users in the capital region of Denmark we compared STI incidence rates and testing frequency before and after initiation of PrEP. We explored the timing of changes in STI incidence related to PrEP initiation, as well as differences between age groups and anatomical site of infections.

### Setting

In Denmark, PrEP has been offered to persons at risk of contracting HIV since 2018. Potential PrEP users are referred to infectious disease departments by general practitioners or by a non-governmental organisation dedicated to preventing HIV and improve sexual health [22]. Requirements for PrEP treatment in Denmark include being (i) HIV-negative, (ii) older than 15 years, (iii) transgender individuals or men who have sex with men (TMSM) engaging in condomless sex with more than one TMSM in the last 12 weeks OR having an STI in the last 24 weeks OR engaging in chemsex OR being sex partner to a person with a non-controlled HIV infection, (iv) having a Danish CPR number and (v) having a normal kidney function. Heterosexual individuals with a high HIV risk may also be eligible. 

PrEP users were screened every 3–6 months for STI. At every visit they were tested for chlamydia and gonorrhoea using swabs from the inside of the mouth and rectum and a urine sample. For syphilis, serological testing was conducted. Except for point-of-care HIV testing, all STI tests are analysed at clinical microbiology departments. Counselling on safe sex practices was provided at PrEP initiation and during follow-ups. Before initiating PrEP, participants were not systematically screened for STIs but were tested as part of the public healthcare services, where tests are easily accessible and provided free of charge. For men who have sex with men with or without symptoms, guidelines recommend urethral, rectal and pharyngeal testing for chlamydia and gonorrhoea [23]. Apart from symptom-based testing, syphilis screening tests are recommended to patients who have being diagnosed with another STI, or to people who engage in unprotected sex [24].

### Study population and data sources

The Danish PrEP Database (DanPrEPD) is an ongoing nationwide prospective cohort study. At enrolment in the PrEP programme, individuals are invited to be part of the DanPrEPD. Upon agreement to enrol, we obtained written consent. In the current study, we included all individuals enrolled in DanPrEPD in the Capital Region of Denmark between 1 January 2019 and 1 June 2022, all of whom belonged to the key population of TMSM. Using participants' CPR numbers, we obtained records of all STI tests (before and after PrEP initiation) from every Clinical Microbiology and Immunology Department in the Capital Region during the study period, ensuring region-wide data collection.

### Outcome

Outcomes were incidence rates and incidence rate ratios (IRR) of chlamydia, gonorrhoea and syphilis, comparing before and after initiation of PrEP. For chlamydia and gonorrhoea, we collected rectal, pharyngeal and genital specimens. When samples from various anatomical sites tested positive on the same date, we considered them as a single STI episode. Regarding syphilis, we identified all positive PCR test from swabs as well as all increases in rapid plasma reagin (RPR) levels, which were subsequently reviewed by medical doctors to either confirm or rule out the diagnosis. As most syphilis diagnoses were based on RPR, which is a blood test, we did not consider anatomical locations for syphilis. We divided participants into age groups based on their CPR number.

### Statistical analysis

The study employed a pre–post design, comparing STI incidence before and after starting PrEP, with participants serving as their own control. We defined the observation time before PrEP from 1 January 2019 to date of PrEP start, and time on PrEP from date of PrEP start until 1 June 2022 or until the participant stopped PrEP. Time after PrEP was defined as from the date the participant stopped PrEP until 1 June 2022. We calculated IRR using negative binominal regression analyses, off-setting for individual observation time. The adjusted IRR controlled for test frequency and age, in line with similar studies [15,21]. We conducted a sensitivity analysis omitting the 6 months preceding PrEP initiation, since having an STI in this period is an indication for starting PrEP [25]. Including this period in the analysis is likely to yield artificially high STI incidence rates due to the selection bias inherent in PrEP indication. In contrast, excluding this period could lead to artificially low estimates, as it actively omits cases, posing a risk of a type 1 error. To balance these considerations, we included the 6-month period in our primary analysis and conducted a sensitivity analysis to evaluate the effects of excluding the period. We used R Studio version 1.4.171725 to perform data analysis and statistical calculations.

## Results

### Participant characteristics

Of the 1,494 participants from the Capital Region enrolled in DanPrEPD during the study period, 1,326 started PrEP and had at least one follow-up STI test. A total of 1,316 (99.2%) participants were self-reported cismen and 10 (0.8%) were transgender or non-binary (Table 1). Age ranged from 16 to 78 years (median: 35 years; interquartile range (IQR): 29–44). While 1,318 individuals (99.4%) were prescribed PrEP as a daily regimen, eight individuals (0.6%) were prescribed PrEP on-demand.

**Table 1:** Participant characteristics, study on gonorrhoea, chlamydia and syphilis incidence among people taking HIV pre-exposure prophylaxis, the capital region of Denmark, January 2019–June 2022 (n = 1,326)

Characteristic	Number	Percentage
Median age in years (IQR)	35 (29–44)	
Cismen	1,316	99%
Daily PrEP regimen	1,318	99%
On PrEP at study start	147	11%
**Mean observation time**	**Years**	**Standard deviation**
Before PrEP	1.70	0.94
On PrEP	1.72	1.05
After PrEP	1.04	0.69

IQR: interquartile range; PrEP: HIV pre-exposure prophylaxis.

### Observation time

Participants were observed for 2,155 person-years before PrEP start and 2,351 person-years after PrEP start. As the observation time was defined by individual PrEP start date, it varied greatly, ranging from 0 to 3.42 years (mean: 1.83 years; standard deviation (SD): 1.05). Notably, 147 participants (11%) were already using PrEP at the beginning of the study, thus their contribution was limited to providing observational data while on PrEP. The after-PrEP group had a much smaller sample size (n = 237 after PrEP compared with n = 1,326 on PrEP) and observation time (247 person-years after PrEP compared with 2,278 person-years on PrEP); we therefore only included this group in descriptive statistics, without calculating IRR.

### Timing of changes in incidence and test rates

**Figure 1:**
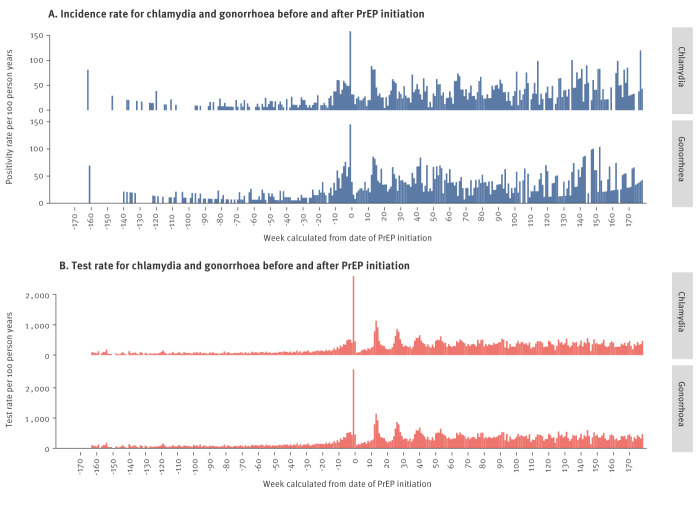
Incidence and test rate for chlamydia and gonorrhoea before and after initiation of HIV pre-exposure prophylaxis, the capital region of Denmark, January 2019–June 2022 (n = 1,326)

Figure 1 shows test rate and incidence per week before and after PrEP initiation. We calculated the rates by dividing the total number of tests by the number of participants who were under observation during that week. As an example, for week −7, the number of positive tests 7 weeks before PrEP start was divided by the number of participants with and observation time of at least 7 weeks before PrEP. The STI test rate and incidence began to rise 10–20 weeks before PrEP start. A large spike in STI incidence and test rates occurred during the PrEP initiation week (week 0) due to screening, which we excluded from the calculations of the incidence rate and IRR. There were peaks in tests frequency around week 13, 26 and 40 after PrEP start, coinciding with trimonthly check-up dates. Notably, data quality was higher for weeks closer to PrEP initiation as more individuals had data available for those periods. Conversely, there were fewer data points further from PrEP initiation because fewer individuals had sufficient data. Finally, due to few events, we did not include a weekly incidence plot for syphilis.

PrEP: HIV pre-exposure prophylaxis; STI: sexually transmitted infections.

The spike in STI incidence and test rates corresponding to the week of PrEP initiation screening (week 0) was excluded from calculations of incidence rate and incidence rate ratios. Due to limited syphilis data, we did not include a weekly incidence plot for syphilis.

### Episodes of sexually transmitted infection

During the observation time before PrEP-start, the incidence rate of any STI (gonorrhoea, chlamydia or syphilis) was 35.3 per 100 person-years (708 STI diagnoses) (Table 2). During PrEP use, the incidence rate rose to 81.2 STIs per 100 person-years (1,849 diagnoses), resulting in an adjusted IRR of 1.35 (95% CI: 1.18–1.56). For chlamydia episodes, the incidence rose from 15.3 (n = 307) to 35.4 (n = 805) with an adjusted IRR of 1.23 (95% CI: 1.03–1.48). For gonorrhoea episodes, incidence was 16.4 (n = 330) before PrEP and 37.4 (n = 850) on PrEP, with an adjusted IRR of 1.24 (95% CI: 1.04–1.47). Finally, for syphilis, incidence rose from 3.5 (n = 71) to 8.8 (n = 194) with a non-significant adjusted IRR of 1.15 (95% CI: 0.76–1.72).

**Table 2:** Incidence rates and test rates for chlamydia, gonorrhoea, syphilis before and on HIV pre-exposure prophylaxis, the capital region of Denmark, January 2019–June 2022 (n = 1,326)

Outcome	Cases before PrEP	Cases on PrEP	IR before PrEP^a ^	IR on PrEP^a^	IRR (95% CI)	Tests before PrEP	Tests on PrEP	Adjusted IRR (95% CI)^b^
Any STI	708	1,849	35.3	81.2	2.15 (1.91–2.41)*	7,936	23,654	1.35 (1.18–1.56)*
Chlamydia								
Any site	307	805	15.3	35.4	2.25 (1.95–2.62)*	3,129	8,202	1.23 (1.03–1.48)*
Rectal	222	569	11.1	25	2.21 (1.86–2.63)*	2,715	7,856	1.26 (1.01–1.56)*
Oral	43	94	2.1	4.1	1.91 (1.32–2.80)*	2,711	7,803	1.19 (0.73–1.92)
Genital	94	274	4.7	12	2.48 (1.90–3.26)*	2,981	7,957	1.14 (0.82–1.60)
Gonorrhoea								
Any site	330	850	16.4	37.4	2.21 (1.91–2.56)*	3,232	8,506	1.24 (1.04–1.47)*
Rectal	212	567	10.6	24.9	2.26 (1.89–2.73)*	2,770	8,057	1.12 (0.90–1.40)
Oral	196	451	9.8	19.8	2.02 (1.68–2.43)*	2,802	8,069	1.16 (0.93–1.46)
Genital	97	189	4.8	8.3	1.66 (1.24–2.23)*	3,011	8,005	0.66 (0.45–0.96)*
Syphilis	71	194	3.5	8.5	2.46 (1.86–3.28)*	1,575	6,946	1.15 (0.76–1.72)

CI: confidence interval; IR: incidence rate; IRR: incidence rate ratio; PrEP: pre-exposure prophylaxis; STI: sexually transmitted infections.

* Statistical significance.

^a^ Per 100 person years.

^b^ Adjusted for differences in test rates and age.

### Sensitivity analysis for indication bias

We performed a sensitivity analysis excluding the last 6 month before individual PrEP initiation. After censoring, the IRR adjusted for age and test rate was 1.91 (95% CI: 1.56–2.35) for any STI, 1.64 (95% CI: 1.26–2.14) for chlamydia, 1.79 for gonorrhoea (95% CI: 1.38–2.32) and 1.83 (95% CI: 1.03–3.26) for syphilis.

### Age stratification

We stratified the data by age categories: under 30 years (n = 449), 30–40 years (n = 529) and over 40 years (n = 483). As the younger group started PrEP later than the older group, they had a longer observation time before PrEP. To adjust for this skewness when comparing age groups, observation time was restricted to 1 year before and 1 year on PrEP. The IRR for the youngest, middle and oldest age groups were 2.09 (95% CI: 1.61–2.71), 2.09 (95% CI: 1.63–2.68) and 2.19 (95% CI: 1.69–2.84), respectively. Due to the restricted observation time, adjusted IRR were not calculated.

### Anatomical site

**Figure 2:**
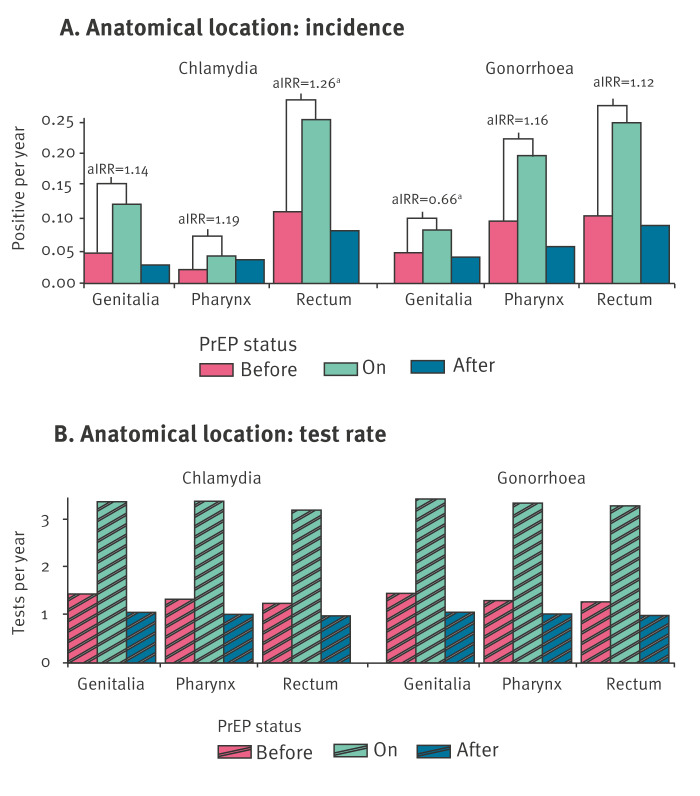
Differences in anatomical sites of sexually transmitted infections, in relation to use of HIV pre-exposure prophylaxis, the capital region of Denmark, January 2019–June 2022 (n = 1,326)

Before participants started PrEP, both rectal and genital samples exhibited a relatively equal distribution of gonorrhoea and chlamydia infections, with rectal infections being the most common (Figure 2). However, in oral samples, gonorrhoeal infection was more common than chlamydia.

aIRR: incidence rate ratio adjusted for test rate and age; PrEP: pre-exposure prophylaxis.

^a^ Statistical significance at 95% confidence level.

We did not calculate aIRR for the after-PrEP group, due to its small size.

For rectal chlamydia, the incidence rate increased from 11.1 cases per 100 person-years before PrEP to 25 cases per 100 person-years during PrEP use, resulting in an adjusted IRR of 1.26 (95% CI: 1.01–1.56). Changes in oral and genital chlamydia were non-significant (Table 2).

For rectal gonorrhoea, the incidence rate increased from 10.6 to 24.9 cases per 100 person-years, resulting in a non-significant adjusted IRR of 1.12 (95% CI: 0.90–1.40). For oral gonorrhoea, the incidence rate increased from 9.8 to 19.8 cases per 100 person-years, with a non-significant adjusted IRR of 1.16 (95% CI: 0.93–1.46). For genital gonorrhoea, the incidence rate increased from 4.8 to 8.3 cases per 100 person years which, when controlling for age and an increased test rate, resulting in an adjusted IRR of 0.66 (95% CI: 0.45–0.96).

## Discussion

In this prospective cohort study, we compared STI incidence before and after the initiation of PrEP. We observed that PrEP use was linked to a more than twofold increase in the incidence of chlamydia, gonorrhoea and syphilis. This finding aligns with previous non-randomised cohort studies [15,26,27], while an earlier randomised study found no association [28], possibly reflecting differences in study design. Furthermore, when we adjusted for the high frequency of STI testing among PrEP users, the incidences of gonorrhoea and chlamydia remained significantly increased, whereas syphilis did not show a significant change. Overall, PrEP was associated with a 35% higher risk of contracting any STI, which is consistent with findings from similar studies [15,27].

Importantly, we found that the increase in STI incidence preceded PrEP initiation by 10–20 weeks, which has not previously been shown. Our results are consistent with an earlier study that observed an increase in STI positivity rates in the months before, but not after, PrEP start [29]. This challenges prior assumptions about the relationship between PrEP usage and STI incidence. The widely used concept risk compensation postulates that PrEP causes increases in STI. However, if risk compensation was valid, we would expect STI incidence to increase when people feel protected against HIV. This increase could occur either immediately following the initiation of PrEP [29], or gradually over time, as they become more assured of the treatment’s protective effect [11]. As no such increase was seen, an alternative interpretation to the association between PrEP and STIs is that changes in sexual risk-taking lead people to PrEP. As the indication for prescribing PrEP is unprotected sex and previous STIs [25], PrEP programmes actively include individuals during periods of their lives when they take sexual risks, for example when becoming part of a sex-positive community, breaking up with a monogamous partner, recently downloading Grindr or for other reasons increasing the risk of contracting an STI. Regardless of the underlying reason behind the STI risk increase, the association between STIs and PrEP does not imply that PrEP causes sexual risk compensation. Rather, it indicates that PrEP is being given when the risk of STIs is increased, i.e. when it is most needed.

We explored whether increases in gonorrhoeal and chlamydial infections affected some anatomical sites more than others. The incidence of oral, genital and rectal chlamydia and gonorrhoea before PrEP initiation, was in line with similar studies involving PrEP candidates [15,21]. On PrEP, rectal and oral infection rates for both diseases were similar, with rectal chlamydia showing a statistically significant increase. However, the overlapping CIs across sites make us cautious about definitive conclusions about relative incidence changes. Notably, the rise in genital gonorrhoea was modest compared with other sites, turning into a net decrease after adjusting for testing frequency. Given that genital gonorrhoea is less likely to be asymptomatic compared with oral or rectal gonorrhoea and chlamydia [30], routine screening may not substantially increase its detection rate; therefore, controlling for test frequency as done in our and similar studies, could potentially result in falsely low IRR. In similar studies, findings have been divergent: one study [27] reported a net decrease in genital gonorrhoea, while another [15] did not, which highlights the need for further investigation. It is crucial that future research on anatomical site differences in STI incidence addresses gonorrhoea and chlamydia separately. Specifically, future studies should consider the reliability of adjusting for test frequency for infections such as genital gonorrhoea that are less often asymptomatic.

Finally, we explored the influence of age on changing STI incidence following PrEP start. There was no statistically significant difference between age groups. Consequently, we cannot recommend that sexual counselling should target a specific age group. Rather, it should be stressed that in Denmark, there is an increase in STIs associated with PrEP start for older as well as younger individuals.

Our study has several limitations. It lacked a control group, behavioural data and compared systematic screening with the ad hoc approach of symptom- and risk-based testing, which complicates the separation of the effects due to changes in sexual behaviour from those arising from increased screening. The lack of randomisation limits a full investigation of the causal relationship between PrEP use and elevated STI incidence. As in other observational studies, the causal relationship risks being confounded by an indication bias. To fully rule out that risk would require a non-anonymised trial, where individuals seeking PrEP are randomised to either participate in the full PrEP programme or receive only screening and counselling without the PrEP medication. However, conducting such a study would today be ethically indefensible, as it would subject participants to an unnecessary risk of contracting HIV. We tried to control for test frequency by including it as a covariate in a negative binomial regression, as was done by Traeger et al. [15], but this approach is limited by the fact that test frequency is not a random variable.

Since STI is one indication for PrEP, the incidence rate in the months before starting PrEP might be artificially elevated, reflecting an indication bias. To address this limitation, we conducted a sensitivity analysis that excluded the observation period 6 months before starting PrEP. This censoring criterion is expected to result in artificially lower incidence rates, as cases are systematically excluded. Therefore, we believe that the true incidence rate lies between the main analysis (including the full observation period) and the sensitivity analysis (excluding the 6-month period).

A limitation in the age group analysis was the uneven balance between time spent on PrEP and time before PrEP initiation. To control for this, we had to limit the observation time, which in turn widened the confidence interval. Furthermore, we lacked data on STI incidence before PrEP start from the 11 % of participants who had already started PrEP before the study began. Finally, we did not have data on the incidence of lymphogranuloma venereum or on STI tests taken in other parts of Denmark or abroad.

A strength of our study was the combination of personal national registration numbers and comprehensive regional databases of microbiological tests, which enabled us to explore the timeliness of increases in STI incidence in relation to PrEP start. The microbiological test database eliminated the need to rely on self-reported data and lowered the risk of skewness due to missing data.

## Conclusion

Our study revealed an increase in overall STI incidence associated with PrEP use. From a public health perspective, it is essential for policymakers to recognise that the association is not indicative of risk compensation. Our findings suggest that individuals frequently seek PrEP during periods when they are at increased risk of contracting STIs. This makes PrEP programmes a critical point of intervention, both for preventing HIV and STIs. For PrEP programmes in similar settings, our findings emphasise the need to go beyond just dispensing medication. It is crucial to provide a safe and supportive environment that includes comprehensive sexual health consultations, along with STI testing, treatment and prevention strategies. Implementing such an integrated approach not only prevents HIV but also enhances overall sexual health.
